# Proteinase-activated receptor-2 (PAR-2) expression on inflammatory cells in severe asthma

**DOI:** 10.1186/1710-1492-10-S2-A50

**Published:** 2014-12-18

**Authors:** Drew Nahirney, Nami Shrestha Palikhe, Cheryl Laratta, Vivek Gandhi, Dilini Vethanayagam, Mohit Bhutani, Irvin Mayers, Lisa Cameron, Harissios Vliagoftis

**Affiliations:** 1 Pulmonary Research Group, Department of Medicine, and Alberta Asthma Center, University of Alberta, Edmonton, AB, Canada

## Background

PAR-2, a G-coupled receptor activated by serine proteinases, is widely expressed in the human body and is involved in inflammation. We have shown that PAR-2 activation in the airways plays a pathogenetic role in mouse models of asthma. PAR-2 expression is increased in the epithelium of asthmatic subjects, but its expression on immune and inflammatory cells of asthmatic individuals has not been. Severe asthma has different phenotypic characteristics from mild-moderate disease. In this study we compared PAR-2 expression on immune cells between subjects with mild/moderate and severe asthma.

## Methods

A total of 36 asthma subjects (24 mild/moderate; 12 severe by ATS guidelines) were recruited at the University of Alberta Hospital and peripheral blood obtained. PAR-2 expression was analyzed by flow cytometry and qRT-PCR in whole blood.

## Results

There were no differences in the % of eosinophils, neutrophils or CD4+ T cells expressing PAR-2 between severe and mild/moderate asthmatics. CD14^high^ monocytes were classified as classical (CD14++CD16-) or intermediate (CD14++CD16+). No difference in total numbers of either monocyte sub-population was noted between the two asthma groups. More intermediate monocytes from patients with severe asthma (33.6±5.1%) expressed PAR-2 compared to patients with mild/moderate asthma (22.4±4.0%, p=0.039), but there was no difference between asthma phenotypes in the percent of classical monocytes expressing PAR-2 (11.7±2.8% vs. 12.5±2.9%). PAR-2 mRNA expression was not different between severe and mild/moderate asthmatics, however, PAR-2 mRNA correlated with total dose of inhaled steroids and inversely correlated with % predicted FEV1.

## Conclusions

PAR-2 expression is increased on intermediate monocytes in subjects with severe asthma. Intermediate monocytes are pro-inflammatory and their numbers are increased in inflammatory diseases. Our data suggest that PAR-2-mediated activation of intermediate monocytes may play a role in the pathogenesis of severe asthma, although the effects of PAR-2-mediated activation of these cells are not known.

